# DPP4 inhibition prevents AKI

**DOI:** 10.18632/oncotarget.20212

**Published:** 2017-08-12

**Authors:** Christoph Reichetzeder, Berthold Hocher

**Affiliations:** Berthold Hocher: Institute of Nutritional Science, University of Potsdam, Germany; Institut für Laboratoriumsmedizin Berlin (IFLb), Germany and Department of Basic Medicine, Medical College of Hunan Normal University, Changsha, China

**Keywords:** acute kidney injury, DPP-4 inhibitors, ischemia reperfusion injury, gliptins, Dipeptidyl peptidase IV

With great interest we have read the publication “Dipeptidyl peptidase 4 inhibitor use is associated with a lower risk of incident acute kidney injury in patients with diabetes” by Chao *et al.*, demonstrating for the first time in a large scale prospectively designed cohort study that the use of DPP-4 inhibitors is associated with a significant risk reduction for the development of acute kidney injury [1]. In a very recently published study we performed a head-to-head comparison of the DPP-4 inhibitors linagliptin, vildagliptin and sitagliptin in a rat model of renal ischemia reperfusion injury [2]. Renal ischemia reperfusion injury was induced in uninephrectomized male rats by clamping the renal artery for 30 min. DPP-4 inhibitors or vehicle were given p.o. once daily on three consecutive days prior to ischemia reperfusion injury and an additional group continued to receive sitagliptin treatment until study end. Results of this study corroborated previous, to some extent inconclusive, evidence of preclinical studies suggesting a beneficial effect of DPP-4 inhibitor treatment in the setting of renal ischemia reperfusion injury. As in preceding studies, no adverse reactions of DPP-4 inhibitor treatment during renal ischemia reperfusion injury were observed. The histological assessment of renal damage revealed ameliorative effects of DPP-4 inhibitor treatment, which were more pronounced if treatment was continued after the induction of renal ischemia reperfusion injury. Similarly, the results of the study by Chao *et al.*, demonstrated an increased risk reduction with higher DPP-4 inhibitor doses. Additionally, our results showed that the treatment with DPP-4 inhibitors led to a significant reduction of osteopontin expression in the kidney of treated rats. This adds to previous evidence demonstrating renoprotection during ischemia reperfusion injury via DPP-4 inhibition by increasing glucagon-like peptide 1 signalling, reducing oxidative stress and inflammatory responses [2]. Another relevant aspect of our study was the observation of pronounced effects of ischemia reperfusion injury on the tubular damage marker osteopontin, whereas only minor effects on cystatin-C, a marker of glomerular function were observed. Furthermore, the histologically assessed extent of renal damage was strongly correlated with plasma osteopontin, but only showed a weak correlation with plasma cystatin-C. Currently, diagnostic classifications of acute kidney injury are based on an impairment of glomerular function, usually measured by the surrogate parameter serum creatinine. However, there is increasing evidence that these clinically established classifications of acute kidney injury might neglect the impairment of tubular function, the actual pathophysiological hallmark of this disease. Future clinical studies of observational and experimental nature should therefore adopt a more detailed biomarker-based characterization of acute kidney injury using markers of glomerular and tubular function. Such a more in depth assessment of acute kidney injury can influence the outcome of studies with clinical endpoints, as emerging evidence suggests [3–5].

The majority of preclinical studies demonstrated efficacy of DPP-4 inhibitors in ameliorating acute kidney injury in non-diabetic settings, considerately different from the results of the study by Chao *et al.* who demonstrated DPP-4 inhibitor associated acute kidney injury risk reduction in a diabetic population. Until now, a variety of preclinical studies, including our current acute kidney injury study and a previous study in non-diabetic CKD, have demonstrated DPP-4 inhibitor efficacy in renal disease in the absence of diabetes, highlighting protective mechanisms beyond blood glucose regulation [2,6]. The combination of preclinical and clinical safety data, clinical data in a large diabetic population, and preclinical efficacy data of DPP-4 inhibitor treatment in non-diabetic acute kidney injury settings, suggests further investigation of DPP-4 inhibitors via randomized controlled trials in non-diabetic subjects at high risk for acute kidney injury. Results of such studies could causally demonstrate that DPP-4 inhibitor treatment might serve as a preventive measure in procedures associated with an increased risk for acute kidney injury.

Taken together, there is considerate preclinical and now also clinical evidence that DPP-4 inhibitor treatment might act beneficial in the setting of acute kidney injury. Exact underlying mechanisms still need more detailed elucidation, as current data points toward various DPP-4 inhibitor mediated favourable effects. Furthermore, randomized controlled trials would still be needed to demonstrate that DPP-4 inhibitor treatment could be used as a preventive pharmacological measure for reducing acute kidney injury during certain high risk procedures.
